# Analysis of angiogenesis related factors in glioblastoma, peritumoral tissue and their derived cancer stem cells

**DOI:** 10.18632/oncotarget.12398

**Published:** 2016-10-01

**Authors:** Alessio D'Alessio, Gabriella Proietti, Gina Lama, Filippo Biamonte, Libero Lauriola, Umberto Moscato, Angelo Vescovi, Annunziato Mangiola, Cristiana Angelucci, Gigliola Sica

**Affiliations:** ^1^ Institute of Histology and Embryology, “A. Gemelli” Faculty of Medicine, Catholic University of the Sacred Heart, Rome, Italy; ^2^ Institute of Pathology, “A. Gemelli” Faculty of Medicine, Catholic University of the Sacred Heart, Rome, Italy; ^3^ Institute of Public Health, Hygiene Division,“A. Gemelli” Faculty of Medicine, Catholic University of the Sacred Heart, Rome, Italy; ^4^ IRCCS Casa Sollievo della Sofferenza, San Giovanni Rotondo, Italy; ^5^ Institute of Neurosurgery, “A. Gemelli” Faculty of Medicine, Catholic University of the Sacred Heart, Rome, Italy

**Keywords:** angiogenesis, glioblastoma, peritumoral tissue, cancer stem cells, hypoxia

## Abstract

The formation of new blood vessels represents a crucial event under both physiological and pathological circumstances. In this study, we evaluated by immunohistochemistry, and/or Western blotting and/or quantitative real time-PCR the expression of HIF1α, HIF2α, VEGF, VEGFR1 and VEGFR2 in surgical glioblastoma multiforme (GBM) and peritumoral tissue samples obtained from 50 patients as well as in cancer stem cells (CSCs) isolated from GBM (GCSCs) and peritumoral tissue (PCSCs) of 5 patients. We also investigated the contribution of both GCSCs and PCSCs on the behavior of endothelial cells (ECs) *in vitro*. Immunohistochemistry demonstrated the expression of angiogenesis markers in both GBM and peritumoral tissue. In addition, *in vitro* tube formation assay indicated that both GCSCs and PCSCs stimulate EC proliferation as well as tube-like vessel formation. An increased migration aptitude was mainly observed when ECs were cultured in the presence of GCSCs rather than in the presence of PCSCs. These findings suggest that relevant neoangiogenetic events may occur in GBM. In particular, VEGF/VEGFR co-expression in PCSCs leads to hypothesize the involvement of an autocrine signaling. Moreover, our results suggest that both GCSCs and PCSCs own the skill of activating the “angiogenic switch” and the capability of modulating EC behavior, indicating that both cell types are either responsive to angiogenic stimuli or able to trigger angiogenic response. Together with our previous findings, this study adds a further piece to the challenging puzzle of the characterization of peritumoral tissue and of the definition of its real role in GBM pathophysiology.

## INTRODUCTION

Among different type of solid tumors, GBM is highly angiogenic and characterized by evident vascular proliferation [1]. In addition, different types of vascularization mechanisms have been described in brain tumors. This includes endothelial cell sprouting from pre-existing vasculature [2], vessel co-option [3] and intussusceptive growth [4]. Vasculogenic mimicry (VM), which refers to the formation of vessel-like structures from lining tumor cells, has been recently correlated to poor survival compared with VM-negative malignant tumors [5, 6]. Our group has been focusing on the potential contribution of the GBM peritumoral compartment [7–10] and its neovascularization [11] which may serve as a potential target for tumor therapy. To this regard, we demonstrated the expression of phosphorylated mitogen-activated protein kinases and of the stem cell marker nestin in the peritumoral tissue of GBM, even in the absence of tumor cells. Moreover, we described that neoangiogenesis occurred in the GBM neighbouring tissue, where nestin and CD105 were expressed in microvessels ECs that showed a morphology quite similar to those present in the tumor. Furthermore, in the peritumoral tissue, pJNK/nestin ratio, as well as the micro-vessel density evaluated by CD105, correlated with the median patient survival time [8, 11], indicating that they may have prognostic implications in GBM patients. Amongst factors that have been shown to affect vessel growth, VEGF (vascular endothelial growth factor) is a central player during both physiological and pathological angiogenesis [12, 13]. It is also well known that developing tumors rapidly deplete oxygen supply, becoming hypoxic. These changes, occurring in the tissue microenvironment, stimulate hypoxia inducible factor (HIF) signaling and VEGF secretion in hypoxia-sensing cells as well as in tumor-associated stromal cells [14, 15], which in turn stimulates tumor vascular growth. In the majority of tumors, only a small fraction of cells (known as CSCs or tumor initiating cells) is capable of initiating a new tumor. These CSCs are characterized by self-renewal capability and resistance to chemo-radiotherapy [16] that make them a potential therapeutic target for the treatment of GBM. Similar to many normal stem cells which reside in the so-called “stem cell niche”, CSCs also rely on an analogous environment, which is thought to be composed of supporting cells, extracellular matrix and other key factors which ensure stem cell viability and maintenance of their characteristics [17, 18]. Interestingly, GBM shows an aggressive behavior invading adjacent healthy tissue and making surgical resection challenging. This phenomenon is presumably due to the presence of CSCs, perhaps located to the margin of GBM [19]. Notably, recurrence that frequently occurs in the peritumoral tissue has indeed shed great attention to this area [20, 21]. It has been also shown that PDGF signaling is relevant for the survival of CSCs derived from the GBM neighboring tissue (PCSCs) and its targeting may be beneficial in the treatment of GBM [22]. Unfortunately, understanding of the precise cellular and molecular mechanisms that regulate the function of cancer-initiating cells in GBM, that may be purposeful in order to design novel therapeutic approaches, is largely unknown. The investigation of the relationship between neural stem cells and ECs may be crucial in GBM, where CSCs closely interact with the vascular niche and promote angiogenesis mostly through the release of VEGF and stromal-derived factor 1 [23, 24]. On the other hand, the definite vascular-rich tumor niche permits the survival of CSCs [17]. More recently, it has been reported the contribution of the Notch ligand, provided by GBM ECs, to the establishment of a stem cell niche where CSCs can self-renew [25]. Notably, it has been demonstrated that although VEGF is one among the major players during blood vessel formation and the expansion of the vascular niche of GBM *in vivo,* it does not directly affect the properties of CSCs [26]. In the present paper, we evaluated and compared the expression of angiogenic markers in GBM and GBM peritumoral tissue as well as in GCSCs and PCSCs derived from them. Moreover, we investigated the capability of both GCSCs and PCSCs to modulate EC properties, such as migration and tube formation *in vitro*.

## RESULTS

### Patient characteristics

The clinicopathological characteristics of the fifty patients recruited in this study are reported in Table 1. Thirty-three patients were males and 17 females; their age ranged from 20 to 78 years (mean: 60.58). All patients had a Karnofsky performance status ≥ 70.

**Table 1:** Clinicopathological characteristics of the 50 adult patients with primary GBM

Patients	Gender	Age at diagnosis (years)	Tumor localization	KPS score	Treatment	Survival time (months)	Clinical outcome
1	M	69	Occipital	80	RT+CH	6	DOD
2	M	67	Parieto-temporal	80	RT+CH	8	DOD
3	M	63	Frontal	100	RT+CH	11	DOD
4	F	47	Frontal	100	RT+CH	14	DOD
5	F	65	Parieto-occipital	80	RT+CH	18	DOD
6	M	70	Temporal	80	RT+CH	32	DOD
7	F	65	Frontal	90	RT+CH	12	DOD
8	M	58	Parieto-temporal	80	RT+CH	19	DOD
9	M	72	Parietal	90	RT+CH	12	DOD
10	M	43	Parietal	80	RT+CH	19	DOD
11	F	62	Temporal	80	RT+CH	17	DOD
12	F	49	Frontal	100	RT+CH	59	DOD
13	F	65	Frontal	90	−	2	DOOC
14	M	69	Frontal	70	−	0,1	DOOC
15	M	72	Fronto-temporal	70	−	2	DOOC
16	M	57	Parietal	100	RT+CH	18	DOD
17	M	64	Frontal	90	RT+CH	11	DOD
18	M	33	Parietal	90	−	0	DOOC
19	F	69	Frontal	70	RT+CH	13	DOD
20	M	61	Fronto-temporal	100	RT+CH	13	DOD
21	F	62	Fronto-temporal	80	RT+CH	14	DOD
22	M	44	Frontal	100	RT+CH	19	DOD
23	F	72	Frontal	80	−	1	DOOC
24	M	68	Temporal	80	RT+CH	3	DOD
25	M	51	Temporal	100	RT+CH	9	DOD
26	M	54	Parieto-occipital	100	RT+CH	15	DOD
27	M	42	Temporal	100	RT+CH	39	DOD
28	M	52	Parieto-temporal	80	RT+CH	13	DOD
29	M	49	Occipital	90	RT+CH	8	DOD
30	F	52	Temporal	90	RT+CH	35	DOD
31	M	20	Parieto-occipital	100	RT+CH	28	DOD
32	M	71	Fronto-temporal	90	RT+CH	5	DOD
33	M	75	Parietal	80	RT+CH	6	DOD
34	F	59	Temporal	100	RT+CH	10	DOD
35	F	67	Temporal	90	RT+CH	23	DOD
36	F	75	Frontal	80	RT+CH	15	DOD
37	M	62	Frontal	100	RT+CH	7	Lost
38	F	71	Occipital	90	RT+CH	9	DOD
39	M	43	Parietal	100	RT+CH	53	DOD
40	M	51	Parieto-temporal	90	RT+CH	38	DOD
41	M	69	Temporal	100	RT+CH	14	DOD
42	M	69	Frontal	80	RT+CH	19	DOD
43	F	53	Temporal	90	RT+CH	12	DOD
44	M	76	Fronto-parietal	80	RT+CH	15	DOD
45	M	78	Frontal	90	RT+CH	2	DOD
46	M	67	Temporal	90	RT+CH	13	DOD
47	F	62	Temporal	80	RT+CH	7	DOD
48	M	72	Temporal	90	RT+CH	6	DOD
49	M	60	Frontal	80	RT+CH	12	DOD
50	F	63	Frontal	90	RT+CH	11	DOD

Abbreviations: M, male; F, female; KPS, Karnofsky performance status; RT, radiotherapy; CH, chemotherapy; DOD, dead of disease; DOOC, dead of other causes.

### Expression of angiogenic molecules in GBM and peritumoral tissue

**Figure 1:**
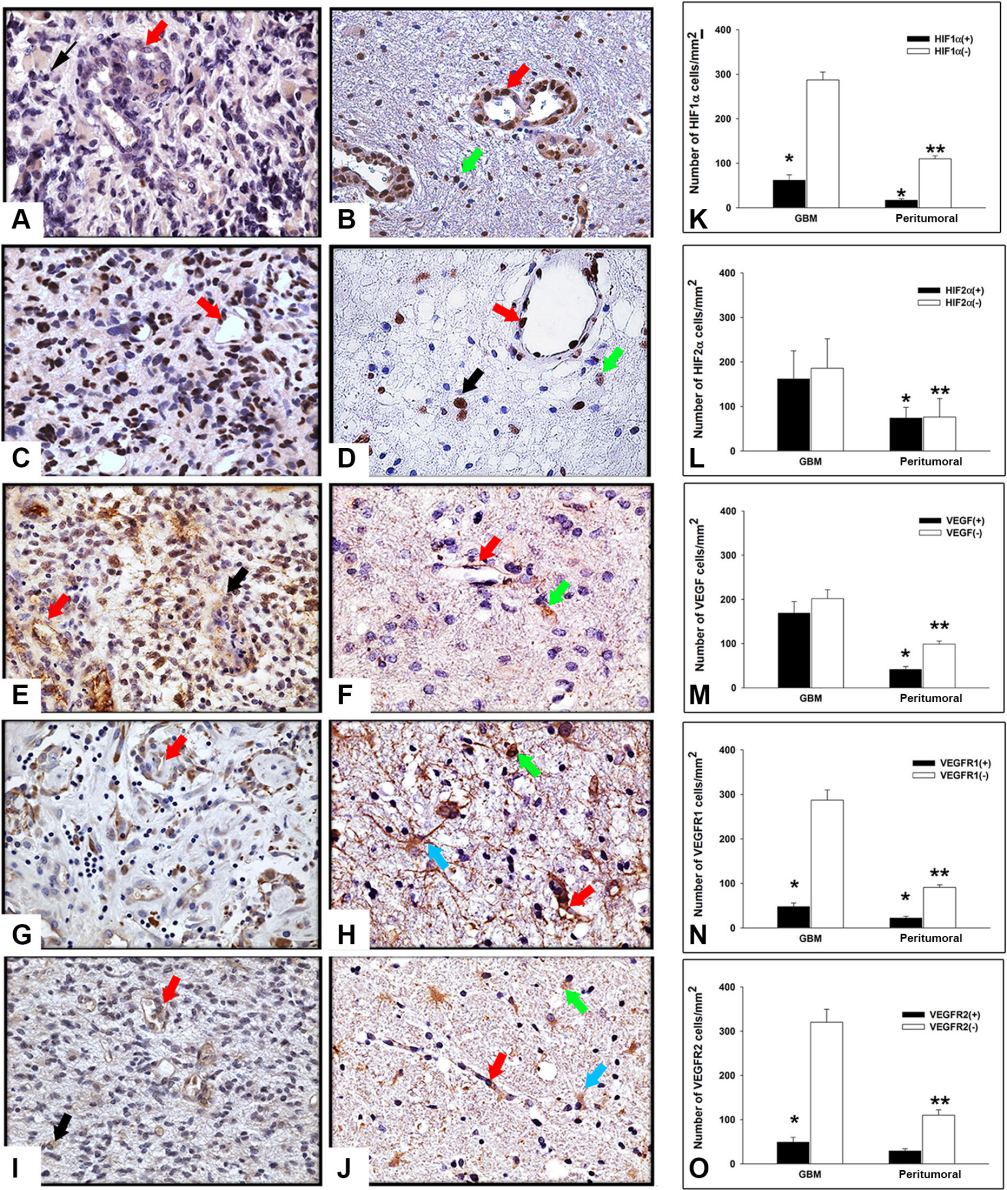
Expression and stereological analysis of angiogenic markers in GBM and peritumoral tissue Immunohistochemistry of HIF1α, HIF2α, VEGF, VEGFR1 and VEGFR2 in GBM and peritumoral tissue. Representative sections from GBM (**A, C, E, G, I**) and peritumoral tissue (**B, D, F, H, J**). (A) In GBM, HIF1α immunopositivity was detected in both the cytoplasm and nuclei of tumor cells (black arrow) and ECs (red arrow). (B) HIF1α immunolocalization in endothelium (red arrow) and in the nuclei of apparently normal cells (green arrow) in peritumoral tissue. (C) HIF2α localization in GBM and (D) in the surrounding tissue was confined in the nuclei of tumor cells (black arrow), ECs (red arrow), and in some apparently normal cells (green arrow). (E) VEGF immunoreactivity appears diffusely distributed throughout the cytoplasm of GBM cells and in endothelium (black arrow and red arrow, respectively). (F) VEGF expression in peritumoral tissue, without tumor cells, in endothelium (red arrow) and in apparently normal cells (green arrow). (G, I) VEGFR1 and VEGFR2 expression was typically located in cytoplasm of tumor cells and ECs (black arrow and red arrow, respectively), as expected. (H, J) An intense specific staining was found in cytoplasm and protrusions of reactive astrocytes (turquoise arrow) as well as in apparently normal cells and endothelium (green arrow and red arrow, respectively). Original magnification X400. Stereological analysis of cell density in both GBM and peritumoral tissue (K–O). Cell density was determined by dividing the cell count by the area of the ROI (*N* = 50). Data shown in the bar graphs are the mean ± ES. Statistical significance was calculated on (+) vs (−) cells and on the peritumoral tissue vs GBM (*p < 0.05; **p < 0.001).

To study the expression of angiogenesis-related molecules we performed immunohistochemistry analysis of HIF1α, HIF2α, VEGF, VEGFR1 and VEGFR2, both in tumor and in peritumoral tissue of GBM surgical samples. As illustrated in Figure 1, immunoreactivity of all the above mentioned markers was detected in the tumor and in peritumoral tissue in different cell types. In particular, HIF1α immunopositivity (Figure 1A) was found in both the cytoplasm and the nuclei of tumor cells and ECs, while in the peritumoral tissue (Figure 1B) the protein was mainly localized in the nucleus of ECs and in some cells with apparently normal morphology. In both GBM and peritumoral tissue, HIF2α expression was confined in the nuclei of tumor cells and ECs (Figure 1C and 1D) and only rare putative normal cells showed an intense nuclear staining (Figure 1D) in surrounding GBM tissue. VEGF immunoreactivity was diffusely distributed throughout the cytoplasm of GBM cells and in ECs, respectively, (Figure 1E) while in the peritumoral tissue, that did not show evidence of tumor cells, VEGF was mainly restricted to endothelium and to a small number of apparently normal cells (Figure 1F). With regard to VEGFR1 and VEGFR2, in GBM samples they were primarily localized to the cytoplasm of tumor cells and in ECs, as expected (Figure 1G and 1I). Moreover, in peritumoral tissue we observed cells, showing a reactive astrocyte morphology, that displayed in the cytoplasmic processes a specific staining for VEGFR1 and VEGFR2 (Figure 1H and 1J). Immunopositivity for these two molecules was also found in apparently normal cells and endothelium (Figure 1H and 1J). Taken together, these results indicated the expression of angiogenesis-related molecules in both GBM and peritumoral tissue. Because of the complexity of tissues studied and in order to exclude biases due to possible human errors during cell counting, we performed a quantitative evaluation of immunostaining using a computer assisted stereoinvestigator, as described in Materials and Methods. First of all, the number of both positive (+) and negative (−) cells for each marker within the region of interest (ROI, 0.035 mm^2^) was investigated. This analysis demonstrated that the number of cells positive for HIF1α, VEGFR1 and VEGFR2 was lower than the number of negative cells (Figure 1K, 1N and 1O; *p* < 0.01, Mann-Whitney test), whereas the number of HIF2α and VEGF positive cells did not significantly differ from that of the negative ones (Figure 1L, 1M). In addition, with the exception of HIF2α (*p* < 0.48), the number of positive cells was significantly lower with respect to the negative stained ones in the peritumoral tissue (Figure 1K–1O). Moreover, our data indicate that the ratio between (+) and (−) cells for HIF1α (Figure 1K), HIF2α (Figure 1L), VEGFR1 (Figure 1N) and VEGFR2 (Figure 1O) was comparable in GBM and in the peritumoral tissue. Conversely, the ratio between (+) and (−) cells for VEGF was significantly higher in GBM (Figure 1M), compared to the peritumoral region of samples obtained from different patients. GBM and peritumoral tissue significantly differed for HIF1α- (Figure 1K; *p* = 0.001, Mann-Whitney test), HIF2α- (Figure 1L; *p* < 0.001, Mann-Whitney test), VEGF- (Figure 1M; *p* < 0.001, Mann-Whitney test) and VEGFR1- (Figure 1N; *p* = 0.013, *p* < 0.001, Mann-Whitney test) positive cell density, with higher values found in GBM than in the peritumoral tissue, while no difference was observed in the density of cells expressing VEGFR2 (Figure 1O; *p* = 0.48, Mann-Whitney test). Interestingly, the density of HIF1α- and HIF2α-positive cells revealed higher expression of HIF2α (Figure 1L) compared to HIF1α (Figure 1K) in both the regions, suggesting different roles for these factors in the regulation of tumor development, as discussed in the next sections. No differences were shown in the expression of the five markers with respect to the presence or absence of tumor cells in peritumoral tissue (data not shown).

### Survival analysis of GBM patients

To evaluate the correlation between the angiogenic markers detected in tissue samples and patient survival, forty-five patients were included in the Kaplan-Meier analysis; the five patients who did not receive radiochemotherapy were excluded (Table 1). This analysis indicated that KPS and gender were not associated with survival time. Furthermore, no association was observed between HIF1α, HIF2α, VEGF, VEGFR1 and VEGFR2 expression in the two areas and survival time. The only parameter which significantly correlated with survival was the age. Patients ≤ 64 years old at diagnosis had a better median survival time compared with patients diagnosed at ≥ 65 years old (14 and 12 months, respectively; *p* = 0.028 log-rank test) (Supplementary Figure S1).

### Expression of angiogenic markers in GCSCs and PCSCs

**Figure 2:**
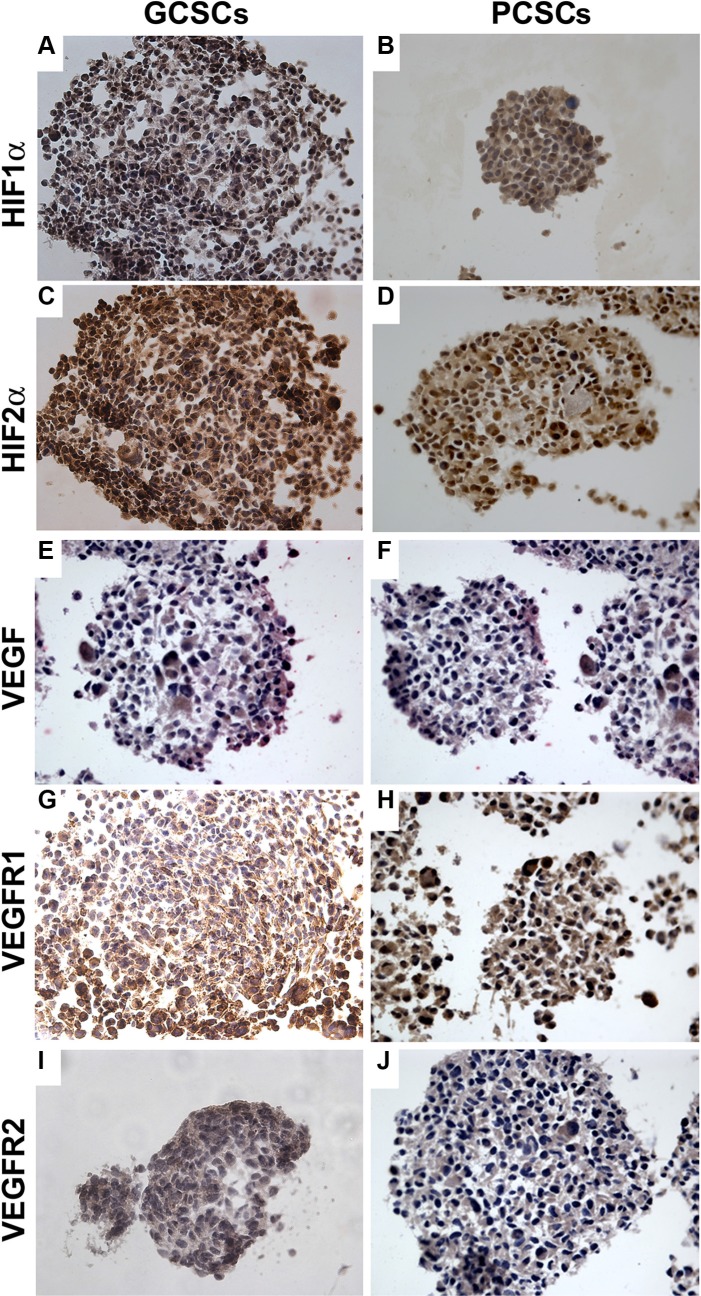
Expression of HIF1α, HIF2α, VEGF, VEGFR1 and VEGFR2 in GCSCs and PCSCs In both GCSCs and PCSCs, HIF1α (**A, B**) and HIF2α (**C, D**) were expressed, and immunoreactivity was detected both in the cytoplasm and in the nuclei. VEGF expression was found in the cytoplasm of both GCSCs (**E**) and PCSCs (**F**). VEGFR1 and VEGFR2 immunopositivity was present in the cytoplasm of GCSCs (**G, I**) and PCSCs (**H, J**). Original magnification x400.

**Figure 3:**
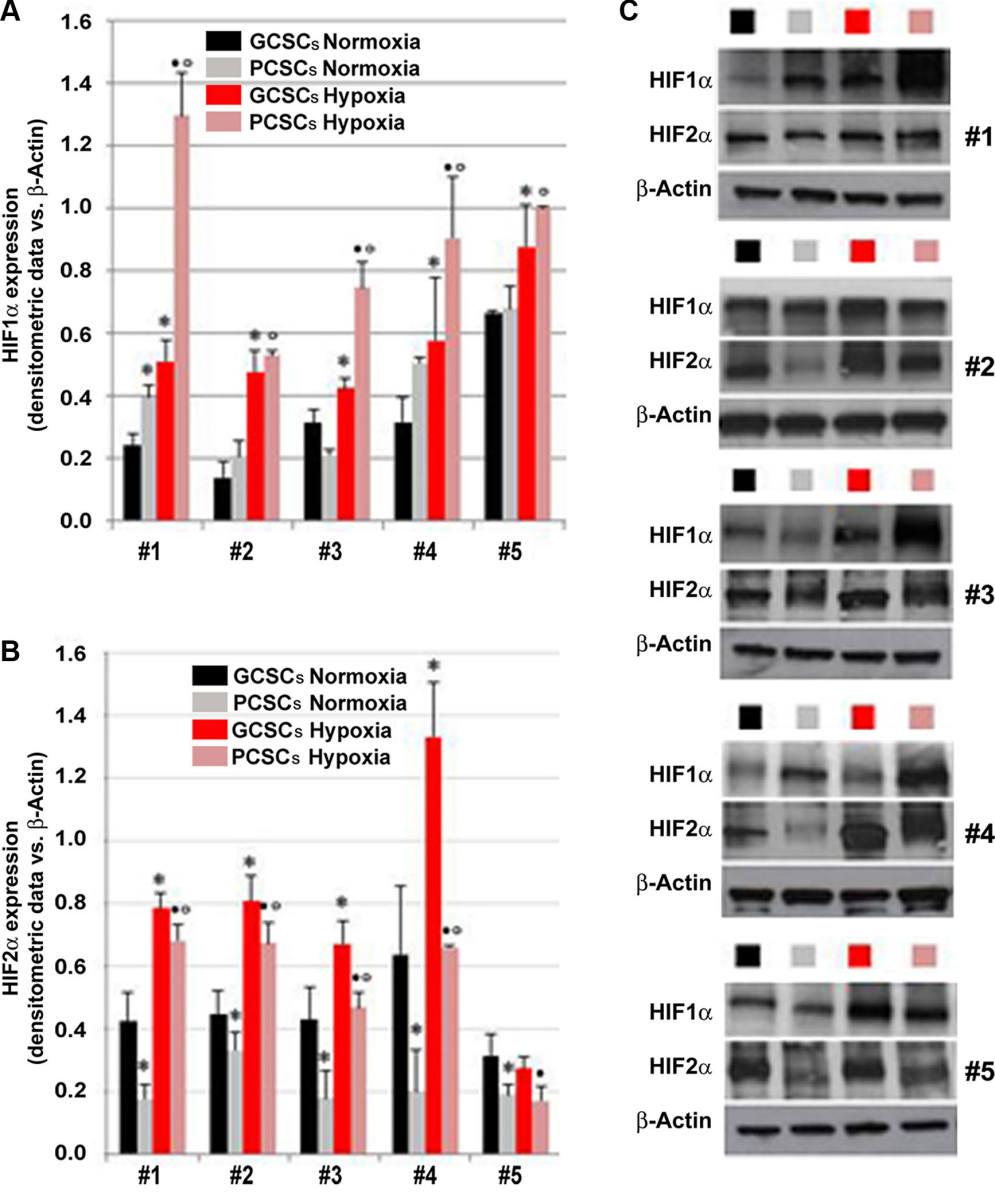
Expression of HIF1α and HIF2α in GCSCs and PCSCs under normoxic and hypoxic conditions Analysis of HIF1α (A and C) and HIF2α (B and C) protein expression by Western blotting in GCSCs and PCSCs of five patients cultured in normoxic or hypoxic conditions. In order to induce hypoxia, a series of GCSCs and PCSCs were treated overnight with 200 μM DFX, a hypoxia-mimicking agent, prior to Western blot analysis. Densitometric analysis (**A** and **B**) revealed an overall hypoxia-triggered increase in the protein level of both the transcription factors that are differentially expressed in GCSCs with respect to PCSCs. (**C**) Representative immunoblots are shown. Data are expressed as densitometric units (normalized to β-actin levels) and are the mean ± SD. *p < 0.05 vs GCSCs normoxia, •*p* < 0.05 vs GCSCs hypoxia and °*p* < 0.05 vs PCSCs normoxia, Student's *t* test.

The expression of HIF1α, HIF2α, VEGF, VEGFR1 and VEGFR2 investigated by immunocytochemistry, was detected in both GCSCs (Figure 2A, 2C, 2E, 2G and 2I) and PCSCs (Figure 2B, 2D, 2F, 2H and 2J). Similar to what was observed in GBM and peritumoral tissue samples, HIF1α and HIF2α showed a cytoplasmic and nuclear immunoreactivity in both GCSCs and PCSCs (Figure 2A–2D), which indicated the activated status of these transcription factors. Moreover, immunocytochemistry analysis highlighted the expression of VEGF (Figure 2E and 2F) and its receptors (Figure 2G–2J). Their immunoreactivity was localized in the cytoplasm and on the cell membrane of GCSCs and PCSCs. Since the HIF pathway is a crucial regulator of angiogenesis and hypoxia regulates a variety of angiogenic pathways that modulate EC functions, we next investigated the expression of HIF1α and HIF2α in GCSCs and PCSCs by immunoblotting under both normoxic and DFX-induced hypoxic conditions which revealed a different behavior of the two transcription factors. Under normoxic condition, HIF1α showed a heterogeneous expression pattern among the GCSC/PCSC couples, with sporadic statistically significant differences in the protein level detected between the two cell populations (Figure 3A). DFX treatment increased HIF1α expression in PCSCs with respect to GCSCs in all samples (Figure 3A and 3C). On the other hand, under normoxic condition, HIF2α was found to be upregulated in all the neurospheres derived from the tumor (GCSCs) with respect to PCSCs (Figure 3B and 3C). The same trend was observed under hypoxic conditions, where, however, a significant increase in HIF2α expression was detected in the majority of the GCSC/PCSC couples with respect to the normoxic state.

### GCSC and PCSC influence on EC behavior

**Figure 4:**
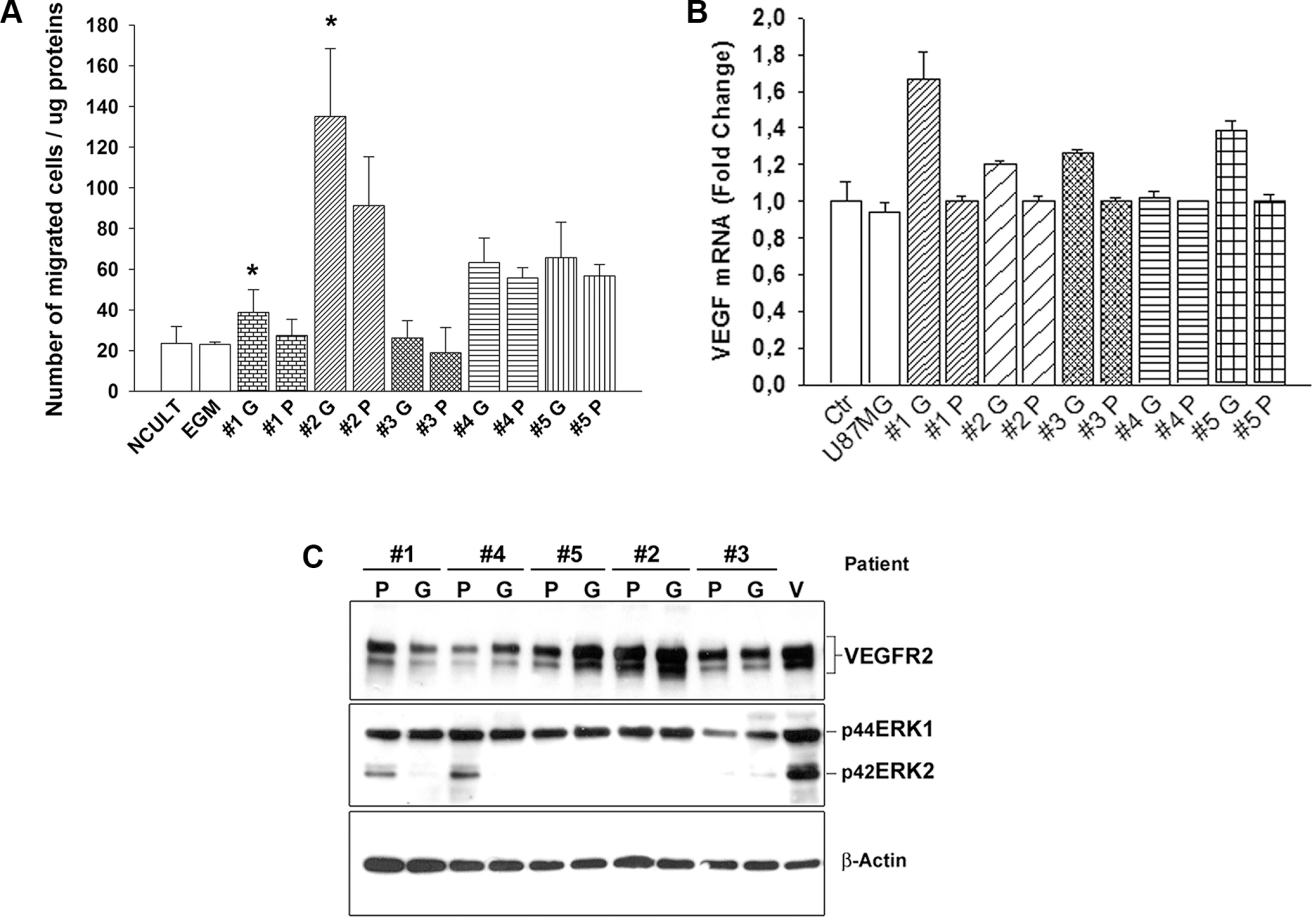
Effect of GCSCs and PCSCs on migration, VEGF gene expression and signaling on HUVECs (**A**) HUVECs were cultured in the presence of conditioned medium harvested from either GCSCs or PCSCs and allowed to migrate for 18 h across the membrane in the Boyden chamber. Cells migrated on the lower surface of the filter were DAPI-stained and counted in 5 fields per well (NCULT, Neurosphere Medium; EGM, Endothelial Growth Medium). (**B**) Evaluation of VEGF mRNA level by qPCR normalized to β-actin. Data show the relative fold change of VEGF transcript in HUVECs co-cultured with GCSCs or PCSCs vs HUVECs (Ctr, set to 1); U87MG glioblastoma cell line was used as an additional control. (**C**) Immunoblotting analysis of VEGFR2 and pERK1/2 expression in HUVECs co-cultured with GCSCs and PCSCs. Representative immunoblots are shown. Values in A and B represent the mean ± SD of three independent experiments. (*p < 0.05 GCSCs vs PCSCs Student's *t-*test). G, GCSCs; P, PCSCs; V, VEGF.

We next aimed to evaluate the specific contribution of GBM- and peritumoral tissue-derived CSCs on EC behavior, *in vitro,* by determining the capability of GCSC- and PCSC-derived conditioned medium to stimulate angiogenic responses. To this regard, since migration of ECs is a crucial mechanism during the angiogenic process, we evaluated the ability of conditioned medium to stimulate the migration of ECs *in vitro*. The Boyden chamber assay demonstrated a greater capability of GCSCs with respect to PCSCs to stimulate the migration of ECs, under this condition, although not all samples of CSCs showed the same amplitude of response (Figure 4A). These results demonstrated the great variability of effects induced by both GCSCs and PCSCs that can be probably blamed on intrinsic differences among patients, as previously reported by others [22]. In addition, these data were supported by the analysis of VEGF mRNA, which was found to be expressed at a higher level in HUVECs co-cultured with GCSCs (Figure 4B), suggesting a potential direct effect of GCSCs, rather than PCSCs, on EC-induced VEGF release. However, in HUVECs co-cultured with either GCSCs or PCSCs, we did not observe any significant difference in the expression of either VEGFR2 or of pERK1/2 (Figure 4C), two well-known factors commonly activated in response to VEGF. In addition, among the two different isoforms of pERK evaluated, the lower one was almost absent in some ECs co-cultured with CSCs. By contrast, VEGF-induced phosphorylation (which served as control) of ERK1/2 showed a similar pattern of both isoforms at 42 and 44 kDa, as expected (Figure 4C). These results indicated that although normal ECs correctly activate ERK pathway in response to VEGF, both GCSCs and PCSCs differently trigger ERK phosphorylation probably by releasing a unique combination of soluble factors, including VEGF.

### *In vitro* evaluation of GCSC- and PCSC-induced tube formation

**Figure 5:**
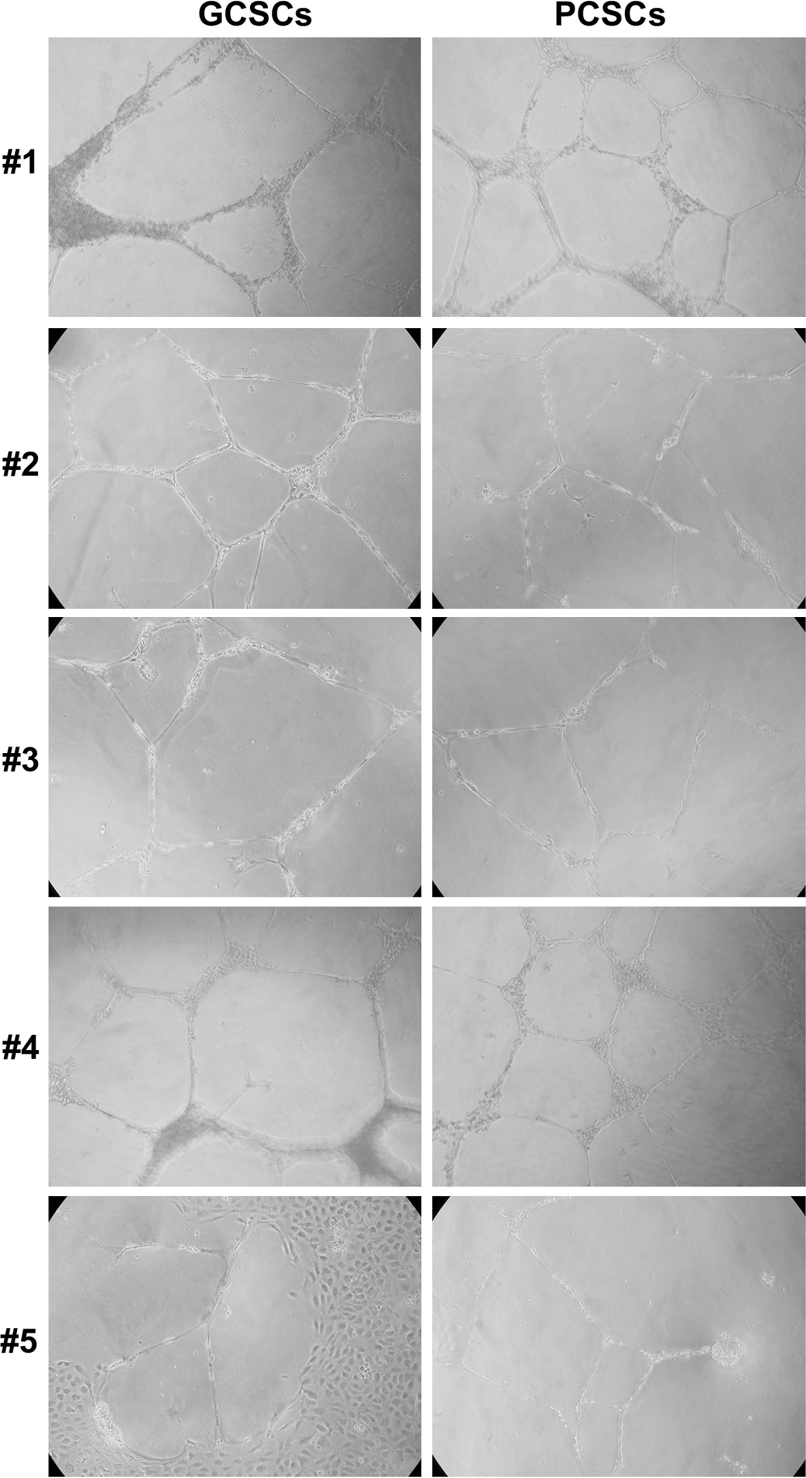
*In vitro* tube formation assay in EC cultivated with GCSCs and PCSCs HUVECs were seeded onto growth factor reduced Matrigel Basement Membrane Matrix and cultured in the presence of GCSCs and PCSCs obtained from five patients (#1–5). Tube formation analysis, evaluated after 18 h of incubation, showed a similar pattern of new vessel organization of EC co-cultured with either GCSCs or PCSCs. Data are representative results of three independent experiments.

Based on the previous observations, we further evaluated the capacity of GCSCs and PCSCs to induce angiogenic responses of ECs, such as the formation of capillary-like tubules *in vitro*. Therefore, an *in vitro* angiogenesis assay was performed by co-culturing either GCSCs or PCSCs with ECs. As shown in Figure 5, our results indicated that both GCSCs and PCSCs similarly induced tube formation *in vitro*. Nevertheless, GCSCs derived from patient #1 and #5, in addition to stimulate tube formation, induced a significant proliferation of ECs as indicated by the presence of a high density area of proliferating cells (Figure 5). These results, together with the ones illustrated previously, highlighted a similar capability of both GCSCs and PCSCs in inducing angiogenic responses in ECs, suggesting that not only tumor but also peritumoral tissue-derived CSCs are endowed with angiogenic resources.

## DISCUSSION

GBM is characterized by a high proliferation rate of tumor cells, diffused necrosis and neoangiogenesis [27]. A number of evidence indicated the presence of infiltrative tumor cells endowed with specific skills able to promote tumor progression in the apparently normal brain tissue surrounding GBM [28–30]. In addition, the interplay between tumor cells and ECs is a crucial event that results in the formation of new blood vessels, promoting tumor progression. The angiogenic dependency of GBM is strongly demonstrated by data from studies with angiogenesis inhibitors, mainly acting against VEGF/VEGFR [31, 32]. The administration of Bevacizumab, an anti-VEGF monoclonal antibody, to GBM patients has been demonstrated to be effective in prolonging both progression-free survival and overall survival. Nevertheless, many patients rapidly develop resistance to anti-VEGF treatments [32]. In a recent study, the dual inhibition of VEGFR and angiopoietin-2 in two orthotopic models of GBM, improved mice survival by reducing cell growth, increasing cell necrosis and promoting morphological normalization of the vasculature [31]. In this study, we first focused on the expression of specific angiogenesis-related markers in GBM, in the peritumoral tissue as well as in GBM- and peritumoral tissue-derived CSCs (defined in this work GCSCs and PCSCs, respectively) of five patients. With regard to tissue samples, since the high structural complexity of GBM and its peritumoral tissue makes cell counting sometimes ambiguous and of difficult interpretation, we turned to a relatively novel approach, namely stereology, in order to accurately estimate the total number of cells expressing each of the angiogenic markers investigated within a region of interest (ROI). This approach allowed us to obtain quantitative and unbiased evidence of a three-dimensional structure, based on the analysis performed on two-dimensional histological sections. By taking advantage of this methodology, we were able to estimate the cell density (cells/mm^2^) of each specific marker by dividing the number of either immunopositive or immunonegative cells by the area of the ROI. Furthermore, we next assessed the ability of GCSCs and PCSCs to affect the behavior of ECs *in vitro*. We report here the expression of HIF1α, HIF2α, VEGF, VEGFR1 and VEGFR2 in both GBM and peritumoral tissue, indicating that both areas contain, to some extent, cells that are either responsive to angiogenic stimuli or able to trigger angiogenic response. These findings are in accordance with and strengthen our previous observation of the presence of the type VI intermediate filament nestin and the proliferation-associated and hypoxia-inducible protein CD105 in the vasculature not only in the GBM but also in the tissue surrounding the tumor [11]. In addition, these data support the occurrence of neoangiogenesis in the peritumoral tissue even in the absence of cells with neoplastic morphology. Although the density of cells positive for each marker resulted highly heterogeneous among patients, our results highlight an overall higher number of positive stained cells in GBM with respect to the peritumoral tissue. However, the high density of HIF2α-positive cells found in both GBM and peritumoral tissue might be indicative of multiple functions of this molecule, such as the maintenance of the CSC population or the induction of a CSC phenotype in non-stem cancer cells [33] and may also indicate the presence of potential hypoxic regions in our tissue samples. Indeed, unlike HIF1α, HIF2α has been markedly reported to be expressed only under elevated hypoxic condition in many organs [34]. Moreover, kinetic of HIF protein activation has been demonstrated to be different, with HIF1α expression reported to be transient and shortly active, while HIF2α activity appears more sustained [34]. However, some studies suggest that the two HIF isoforms may play differential and non-overlapping roles in cancer due to their unique target genes as well as dissimilar responses to restricted oxygen levels. Since the expression of HIF1α or HIF2α has been detected at higher level in tumor tissues compared to the tumor surrounding tissue, we can hypothesize that the high expression of HIF2α in peritumoral tissue might be seen as a further sign of an initial transformation. Regarding the expression of VEGF and its receptors, in agreement with previous studies [35, 36] we found VEGF considerably higher in the tumor, with almost 50% of the cells expressing this marker, with respect to the peritumoral tissue. By contrast, the cellular density of VEGFR1 and VEGFR2 was low in both regions. However, it has been also reported that the expression of VEGF in different type of tumors does not necessarily correlate with the expression of its receptors [37]. Moreover, it has been recently described a heterogeneous expression pattern of VEGF as well as VEGFR1 and 2 among astrocytomas of different grades and that beneficial response to Bevacizumab treatment is independent of the expression of VEGF and its co-receptors [38]. Our *in vitro* studies on GCSCs and PCSCs indicate the hypoxia dependency of HIF factors. In particular, the data concerning the expression pattern of HIF1α confirmed the reliance of this transcription factor on oxygen depletion. The increased levels of this factor found in PCSCs may suggest a possible role in the development of the resistance to chemo/radiotherapy occurring after the complete surgical removal of the GBM enhanced lesion. Indeed, high HIF1α levels were previously demonstrated to be responsible for the induction of MDR1 gene and resistance to chemotherapy in HIF1α-transfected human hepatoma cells [39] as well as in human cervical cancer cells [40]. With regard to the expression of HIF2α in GCSC and PCSC neurospheres, the higher protein levels found in the former cell population was also in agreement with the expression of other angiogenesis related molecules such as NG2 and VEGFR1, which are targets of HIF2α [10, 41, 42]. This finding supports the idea of a more defined stem nature for GCSC population and it is not surprising given the role of HIF2α in maintaining hypoxic tumor cells in an undifferentiated and malignant state. The high expression of VEGF observed in GBM tissue can also explain the increased migration aptitude of ECs cultured in the presence of GCSCs rather than PCSCs. However, we observed an unusual activation of pERK1/2 signaling in ECs cultured with either GCSCs or PCSCs, suggesting that a unique combination of soluble factors, including VEGF, are released by CSCs. Interestingly, *in vitro* tube formation assay indicated that both tumor and peritumoral tissue-derived CSCs can activate angiogenesis and in some instances proliferation of ECs, even though to different extents. These results indicate that both GCSCs and PCSCs own the skill of activating the “angiogenic switch”, a critical control point for tumor growth, when tumor begins to overexpress pro-angiogenic factors. Whether or not the tumor recruited newly-born vessels were more permeable in response to soluble factors, such as VEGF, released by GCSCs or PCSCs, has not been investigated in this work. The precise nature and combination of factors released by GCSCs and PCSCs that can affect tumor blood vessel formation is still unclear, encouraging further efforts in the investigation of the mutual interaction between ECs and other cell types within GBM microenvironment. Since the main issue with the anti-angiogenic therapies (e.g. Bevacizumab) is the lack of biomarkers and angiogenic profiles which allow identifying patients who may benefit from this kind of treatment [43], it is important to better characterize the angiogenic signature of GBM and peritumoral tissue in order to identify other effective targets that may improve the management of GBM. In conclusion, taken together with our previous published data [7–11], the present findings strongly suggest the occurrence of early tumorigenic events in the GBM neighbouring tissue as well as the involvement of CSCs residing in the peritumoral niche in the GBM radio- and chemo-resistance, which eventually results in tumor recurrence. This might prefigure a possible role of PCSCs as therapeutic target.

## MATERIALS AND METHODS

### Patients and tissue samples

Primary tumor specimens and adjacent brain tissue were obtained from 50 patients diagnosed with primary supratentorial GBM who underwent “en bloc” surgery at the Institute of Neurosurgery, Catholic University of the Sacred Heart in Rome. GBMs were considered as “primary” based on the patients' clinical history, very short time-interval between first symptom-signs of disease, admission to hospital and a rapid clinical progression. Neuronavigation and intraoperative ultrasound were used to define and maximize the extent of intracranial tumor resection. Tumor and neighboring apparently normal tissue were removed “en bloc”. The surgical specimens were cut and opened in a “book-wise” fashion. Using this technique, the difference between tumor border and its surrounding apparently normal white matter was evident and the distance from white matter adjacent to tumor edge and this latter was well defined and measured [44]. In each patient, the complete removal of the enhanced lesion (T1-weighted zone) and the extension of the resection till the on T2-weighted MRI zone was confirmed by an early contrast MRI (within 24/48 h after surgery), comparing pre- and post-operative contrast-enhanced images. Paired GBM and peritumoral tissue samples were obtained from the enhanced lesion without areas of necrosis (GBM) and from the white matter at a distance < 1 cm from the macroscopic tumor border (peritumoral tissue). None of the patients had received radiotherapy or chemotherapy before surgical resection. Thirty-five to forty days after surgery, all patients underwent irradiation and chemotherapy performed according to literature [45]. The histological diagnosis was evaluated for sections stained with H&E according to the 2007 World Health Organization (WHO) classification guidelines [1]. Neoplastic cells were identified by their nuclear atypia and heteropycnotic staining. Reactive astrocytes were recognized by dendritic morphology of their abundant eosinophilic cytoplasm and large eccentric nuclei [46]. The survival period was defined as the time elapsed from the date of surgery to the date of death. All patients provided informed written consent to use tumor, peritumoral tissue as well as clinical data. The use of human tissues was approved from Ethics Committee of the Catholic University of the Sacred Heart, Rome, Italy (Prot. A/205/2011).

### Cell cultures

Fresh surgical GBM and peritumoral specimens (at a distance <1 cm from macroscopic border), derived from five patients, were dissected and digested in papain solution (Worthington Biochemical, Lakewood, NJ, USA). The neurosphere cells, termed in this work Glioblastoma Cancer Stem Cells (GCSCs) and Peritumoral Cancer Stem Cells (PCSCs) were isolated, cultured and maintained in NeuroCult™ NS-A Proliferation Kit (Stemcell Technologies Inc, Vancouver, BC, Canada) supplemented with 20 ng/ml human recombinant EGF, 10 ng/ml human recombinant bFGF and 2 ug/ml heparin (all from StemCell Technologies Inc.), as described previously [47, 48]. Commercially available pooled Human Umbilical Vein Endothelial Cells (HUVEC) were obtained from Lonza Sales Ldt (Switzerland), cultured in EGM-2 Endothelial Cell Growth Medium-2 (Endothelial Basal Medium EBM-2 + EGM-2 Bullet Kit, Lonza), supplemented with 100 units/ml penicillin/streptomycin (Life Technologies Corporation, Gaithersburg, MD, USA). U87MG grade IV glioma cell line was kindly provided by Dr. Emilio Ciusani, (“Carlo Besta” National Neurological Institute, Milan, Italy) and maintained in DMEM containing 10% (v/v) fetal calf serum, 200 mM L-glutamine, 100 units/ml penicillin/streptomycin (Life Technologies). All cell types were maintained at 37°C in a 5% CO_2_ humidified atmosphere.

### Antibodies and chemicals

For immunohistochemistry, primary antibodies against HIF1α (rabbit monoclonal, clone EP1215Y, Millipore, Ca, USA), HIF2α (rabbit polyclonal, Novus Biologicals, Littleton, CO, USA), VEGF (mouse monoclonal, clone VG1, DakoCytomation, Carpinteria, CA, USA), VEGFR1/Flt-1 (goat polyclonal, R&D Systems, Minneapolis, MN, USA) and VEGFR2/KDR (mouse monoclonal, clone 89115, R&D Systems) were used.

For Western blot analysis, primary antibodies against VEGFR2 (rabbit monoclonal, clone 55B11) and phosphorylated (p) ERK1/2 (mouse monoclonal, clone E10) and the recombinant human VEGF_165_ were purchased from Cell Signaling Technologies (Danvers, MA, USA); anti-β-Actin monoclonal antibody (clone AC15) was from Sigma (Sigma-Aldrich, St. Louis, MO, USA); rabbit monoclonal anti-HIF1α, clone EP125Y, was from Millipore; rabbit polyclonal anti-HIF2α was from Novus Biologicals. Deferoxamine mesilate (DFX) was from Sigma (Sigma-Aldrich).

### Immunohistochemical analysis

Paraffin-embedded tissue sections (5-μm thick), from tumor and peritumoral samples were used for immunohistochemistry analysis. Immunostaining for HIF1α, HIF2α and VEGF was performed using the standard protocol on automated staining Dako Autostainer PlusLink (Dako, Glostrup, Denmark). Target Retrieval Solution (pH 9) was utilized for antigen unmasking on the Dako PT Link Autostainer. The following primary antibodies anti-HIF1α (1:250); -HIF2α (1:300); -VEGF (1:50) were used. The signal was detected and visualized using the EnVision^TM^ +Dual Link System-HRP/DAB, Rabbit/Mouse Kit (Dako). A manual staining method was used to reveal VEGFR1 and VEGFR2 expression. Slide-mounted sections were deparaffinized and rehydrated and antigen retrieval was performed in a microwave oven at 500W for 3 min in 10 mM sodium citrate buffer (pH 6.0). Hydrogen peroxide (0.3%) was applied to block endogenous peroxide activity. Block of non-specific staining (Super Block, UCS Diagnostic S.r.l., Morlupo, Italy) was followed by incubation with primary antibodies against VEGFR1/Flt-1 (1:20) and VEGFR2/KDR (1:20). Subsequently, the sections were incubated with HRP/Fab polymer conjugate (SuperPicTure Polymer DetectionKit, Invitrogen, Camarillo, CA, USA). The location of the reaction was visualized with 3,3′-diaminobenzidine (Peroxidase DAB substrate Kit, Vector Laboratories Inc., Burlingame, CA, USA) resulting in the expected brown colored signal. Finally, all the sections were counterstained with Mayer's hematoxylin and dehydrated in ethanol before mounting. No staining was detected in negative control sections in which primary antibody has been omitted. For an accurate quantification of immunopositive cells, we employed an unbiased stereological technique by means of the Stereo Investigator system (Stereo Investigator software, Version 9.14© 2010, MicroBrightField Europe, Magdeburg, Germany) [49, 50]. A stack of MAC 6000 controller modules (Ludl Electronic Products, Ltd., Hawthorne, NY, USA) was configured to interface a light microscope (Nikon Eclipse 80i, Nikon Corporation, Tokyo, Japan) with a motorized stage and a color digital camera (MicroBrightField) with a PC workstation. First, each region of interest (ROI) was outlined at low magnification (x100) and then scanned using the ‘Meander scan’ function. All immunopositive and immunonegative cells within ROI were counted at x400 magnification. Cell density was determined by dividing the number of immunopositive or immunonegative cells by the area of the ROI (cells/mm^2^).

### Immunocytochemical analysis on GCSCs and PCSCs

GCSC and PCSC neurospheres from five patients were fixed in 4% paraformaldehyde, cryoprotected in 30% sucrose, snap-frozen in liquid nitrogen and stored at −80°C until use. Frozen sections (10 μm) were evaluated by immunocytochemical staining for the expression of HIF1α (1:100), HIF2α (1:100), VEGF (1:100); VEGFR1/Flt-1 (1:100) and VEGFR2/KDR (1:100), as described in the immunohistochemistry procedure (see “Immunohistochemical analysis”). In all immunostaining experiments, negative controls were performed by omitting the primary antibodies.

### Quantitative real time PCR analysis (qPCR)

The expression of VEGF mRNA was evaluated by qPCR analysis. Total RNA was extracted with the TRIZOL Reagent (Life Technologies Corporation), according to the manufacturer's instructions. Therefore, 3 to 5 μg of total RNA were retro-transcribed into single-stranded DNA by a standard 20 μl RT reaction with the High Capacity cDNA Reverse Transcription Kit (Applied Biosystems, Foster City, CA, USA). Real time quantitative RT-PCR was performed using the Step One Real-Time PCR System (Life Technologies Corporation). cDNA generated from the reverse transcription reactions was amplified by PCR with the SensiMix SYBR kit (Bioline, London, UK) in a total volume of 20 μl, according to the manufacturer's instructions. The primers used were as follows: VEGF, 5′-TGAGCTTCCTACAGCACAAC-3′ and 5′-ATTTA CACGTCTGCGGATCTT-3′; β-Actin, 5′-TGCACCACACC TTCTACAATGA-3′ and 5′-CAGCCTGGATAGCAACGT ACAT-3′. The level of gene expression was expressed as relative fold change vs the β-Actin mRNA using the ΔΔ_Ct_ method [51] using the Step One System Software (Life Technologies Corporation).

### Western blot analysis

For immunoblotting analysis, cells were harvested and lysed in lysis buffer containing 50 mM Tris-HCl (pH 7.6), 150 mM NaCl, 1% Nonidet P-40, 0.5% Triton X-100, 0.5 M EDTA and 0.1% SDS, containing complete protease inhibitors cocktail (Sigma-Aldrich) for 15 min at 4°C. Protein concentration was determined by Bradford Protein Assay (Bio-Rad Laboratories Inc, Hercules, CA, USA) according to the manufacturer's instructions. Equal amounts of proteins were then separated by SDS/PAGE (4–20% Mini-PROTEAN^®^ TGX™ Precast Protein Gels, Bio-Rad Laboratories) and transferred to a nitrocellulose membrane (GE Healthcare, Piscataway, NJ, USA). Membranes were blocked with TBS-T (20 mM Tris-HCl, pH 7.4, 137 mM NaCl, and 0.1% Tween-20) containing 5% nonfat milk for 1 h at room temperature (RT) prior to incubation with primary antibodies (anti-HIF1α, 1:1000; anti-HIF2α, 1:500; anti-VEGFR2, 1:1000; anti-pERK1/2 1:1000, anti-β-actin, 1:10000), followed by incubation with horseradish peroxidase conjugated secondary antibody (Vector Laboratories, Burlingame, CA, USA) for 1 h RT. Protein bands were visualized on X-ray films (Hyperfilm ECL, GE Healthcare) using an enhanced chemiluminescence system (SuperSignal Chemoluminescent substrate, Thermo Fisher Scientific Inc. Waltham, MA, USA). The intensity of specific bands was normalized to the intensity of the corresponding β-actin bands. In order to induce hypoxia, a series of GCSCs and PCSCs were treated overnight with 200 μM DFX, a hypoxia-mimicking agent, prior to Western blot analysis.

### Evaluation of EC migration and tube formation assays

Cell migration assay was performed using the modified Boyden chamber assay with an 8 μm polycarbonate membrane inserts (BD Biosciences, Bedford, MA, USA). A suspension of 150,000 HUVECs was added to the upper well of the Boyden chamber and 400 μl of GCSC- or PCSC-derived conditioned medium was added to the lower well. Migration was allowed to proceed for about 18 h in a 37°C, 5% CO_2_ incubator. At the end of the experiment, membranes were washed in PBS and fixed by 3.7% formaldehyde for 10 min at RT and permeabilized by 100% methanol for 20 min at RT. Non migrated cells were gently scraped off with cotton swab and migrated cells were stained with DAPI and counted under a fluorescent microscope (Nikon Eclipse TS100, Nikon, Tokyo, Japan). DAPI-stained filters were quantified by counting five high-power microscopic fields (X40 objective lens) on each well. The number of migrated cells was further normalized to total protein content of GCSC or PCSC conditioned medium. Tube formation was evaluated by angiogenesis *in vitro* assay [52]. Briefly, 150 μl of growth factor reduced Matrigel Basement Membrane Matrix (BD Biosciences) was added onto pre-cooled 48-well tissue culture well plates and left to solidify at 37°C for 60 min. HUVECs (40,000 cells/well) were seeded onto the bottom well of a Boyden chamber containing the polymerized matrix and co-cultured in the presence of either GCSCs or PCSCs, plated on the upper chamber containing a 0,4 μm polycarbonate membrane insert, or stimulated with 100 ng/ml human recombinant VEGFA_165_ (R&D Systems) for about 18 h at 37°C. Tube formation was analyzed under an inverted microscope at 20X magnification by evaluating the formation of polyhedral closed structures delimiting a lumen and images were acquired by a digital camera (Nikon Coolpix995).

### Statistical analysis

Statistical analysis was carried out using SPSS software package for Windows (version 22.0.1 SPSS, Inc., Chicago, IL, USA). Since the expression levels of variables considered, in tissue samples, were not normally distributed, the Mann-Whitney non-parametric test was applied to find statistically significant differences between groups related to the expression of the 5 markers examined (HIF1α, HIF2α, VEGF, VEGFR1, VEGFR2) in GBM and peritumoral tissue. The same test was also applied in order to investigate differences in the expression of the five markers with respect to the presence or the absence of cancer cells in peritumoral tissue. As far as survival analysis was concerned, 6 observations were removed (5 because patients did not receive chemotherapy and radiotherapy, 1 because the patient was lost at the follow-up). Survival analysis was carried out with respect to death. The relationship between the expression of the above mentioned molecules and survival were analyzed. In particular, the median value of expression in the two tissue areas was used as a cut-off point to dichotomize the patients in two groups. Age and KPS (Karnofsky Performance Status) were classified as follows: age (≤ 64 = 0; ≥ 65 = 1), KPS (70–80 = 0; 90–100 = 1). Kaplan-Meier curves together with Breslow and log-rank test were used in order to perform univariable analysis with respect to qualitative variables. To evaluate the significance of the differences in Western blot and migration experiments two-tailed Student's *t*-test was used. An alpha level of less than 0.05 was used for statistical significance in all tests.

## SUPPLEMENTARY MATERIALS


