# Supplementary material for: Analysis of angiogenesis related factors in glioblastoma, peritumoral tissue and their derived cancer stem cells
Source: Oncotarget. 2016 Oct 1;7(48):78541–56. doi: 10.18632/oncotarget.12398 (PMC5346658; doi:10.18632/oncotarget.12398)
Supplement: Supplementary file 1 [file oncotarget-07-78541-s001.pdf]

Analysis of angiogenesis related factors in glioblastoma, peritumoral tissue and their derived cancer stem cells

Supplementary Materials

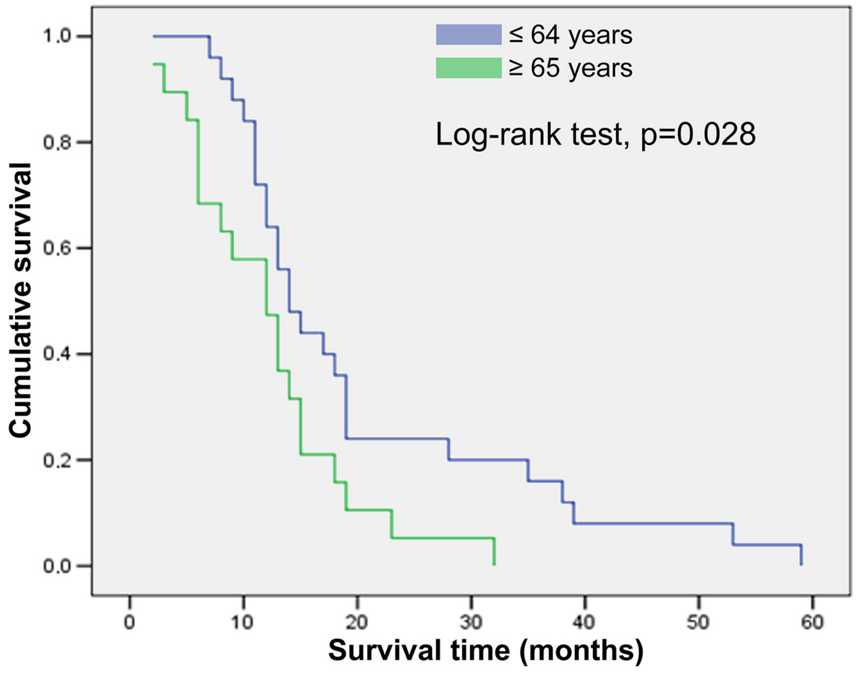

Supplementary Figure S1: Kaplan-Meier plot. The Kaplan-Meier plot depicts that patients ≤ 64 years old at diagnosis had a better survival compared with patients diagnosed at ≥ 65 years old.
